# Supplementary material for: “Are they trying to control us people?”: News media coverage of COVID-19 lockdown tobacco sales ban in South Africa
Source: PLoS One. 2022 Dec 12;17(12):e0278888. doi: 10.1371/journal.pone.0278888 (PMC9744309; doi:10.1371/journal.pone.0278888)
Supplement: Attachment — Submitted filename: Response to the reviewers_26Aug22.docx [file pone.0278888.s003.docx]

**Response to the reviewers**

**[PONE-D-22-09801R1]**

**“Are they trying to control us people?”: News media coverage of COVID-19 lockdown tobacco sales ban in South Africa**

**Journal Requirements:**

**Please review your reference list to ensure that it is complete and correct. If you have cited papers that have been retracted, please include the rationale for doing so in the manuscript text, or remove these references and replace them with relevant current references. Any changes to the reference list should be mentioned in the rebuttal letter that accompanies your revised manuscript. If you need to cite a retracted article, indicate the article’s retracted status in the References list and also include a citation and full reference for the retraction notice.**

**Authors’ response:** We have not used any retracted paper in our manuscript. We wish for the reviewer to mention the paper he or she thinks or knows has been retracted so that we can remove such paper(s) accordingly.

The retracted article of Stanton Glantz referred to by the reviewer is: *Bhatta DN, Glantz SA. Electronic Cigarette Use and Myocardial Infarction Among Adults in the US Population Assessment of Tobacco and Health. J Am Heart Assoc. 2019;8:e012317. DOI: 10.1161/JAHA.119.012317.* Please see link to retraction statement <https://www.ahajournals.org/doi/10.1161/JAHA.119.014519>

In none of the submitted versions of our paper have we mentioned or cited this article. The only two articles co-authored by Prof Stanton Glantz cited in our paper are:

*39. Crosbie E, Sebrié EM,* ***Glantz SA****. Tobacco industry success in Costa Rica: the importance of FCTC article 5.3. Salud publica de Mexico. 2012;54(1):28-38.*

*40.	Crosbie E, Sebrié EM,* ***Glantz SA****. Strong advocacy led to successful implementation of smokefree Mexico City. Tob Control. 2011;20(1):64-72.*

These papers have nothing to do with e-cigarettes or harm reduction. Is the reviewer also calling for these articles to not be used because they were co-authored by Stan Glantz? Prof. Glantz has authored thousands of papers but the reviewer’s attempt to discredit his works because of a retracted paper is not supposed to be done through the review of our paper. This somewhat reads more like a personal vendetta.

**6. Review Comments to the Author**

**Please use the space provided to explain your answers to the questions above. You may also include additional comments for the author, including concerns about dual publication, research ethics, or publication ethics. (Please upload your review as an attachment if it exceeds 20,000 characters)**

**Reviewer #2: I do not feel the most important comment about the harm reduction continuum and use of much less harmful non combusted consumer products (not just NRT) was addressed in the revision. at the very least a coup[le sentences in support of harm reduction and stronger citing of balanced literature rather than the selective use of very weak science and misleading statements by Dr Standton Glants and co authors are NOT a good reason to exclude some framing of the potential for non combusted nictine delivery - along the harm reduction continuum --to be used as a way to make combusted smoking pbsolete more rapidly and pragmatically. This is not beyond the scope of this paper at all and should be included. Moreover the authors seem to themselves adhere to the misinformation promulgated by the weakest science against e-cigarettes (vaped nicotine) and selectively ignore the much stronger scientific evidence in support of harm reduction as a pragmatic solution to the obvious failure of different attempt at total nicotin e prohibition (an extremist view that may be both unrealistic and unreachable -- and totally relevant to the main focus of this paper.**

**The UK approach and Public Health England's excellent reviews and updates as well as stronger balances science from the USA cannot simply be dismissed as beyond the scope of this paper -- indeed that is the main GOAL of the public health approach at the population level -- elimination of the largely preventable deaths and massive chonic disease burdens of decades of COMBUSTED SMOKE INHALATION and toxins in burned tobacco and not of nicotine when delivered in non-combusted but appealing forms other than medicinal NRT where there is clear evidence of much lower levels and much fewer types of toxins in e-cigarette vapor , and in use of Swedish type snus and newer nicotine pouches and so forth. There is ample evidence of dramatic reductions in biomarkers of harm especially major cancer bio-makers such as from FDAs own PATH analysis of toxins in e-cigarette users blood versus combusted users . the major USA Institute of Medicine, the FDAs own 2017 strategy for managing nicotine (Gottlieb and Zeller ); several well done independent reviews ; many of the best studies of exposure and many critical comments about the limitations of poorly done studies that are often used to mislead. Including one study by Glantz et al -- on e-cigarettes and heart attacks --that had to be and was withdrawn by the journal (Am Heart Assn) and others that should have been withdrawn.**

**Authors’ response:** We believe the particular mention of an author as a target of criticism as feedback for our paper when we did not use his retracted work (among the thousands he has authored) is not scientific and should not be part of the review process. We believe our work should be reviewed based on what the scope of this study was. Our study was neither one of smoking cessation nor harm reduction but of media reports of issues discussed in the public domain through the media in South Africa during the tobacco lockdown.

We are worried that this reviewer is explicitly requiring us to support his own stance on e-cigarettes as a harm reduction tool rather than pay heed to a more balanced review of the issue. The condemnation of other scientific evidence of the harms caused by e-cigarettes shows that the reviewer is not willing to objectively review the harms continuum that s/he is suggesting. However, we still believe that our paper is not the place to make a case for harm reduction or not, as we only aimed to objectively present what local media reported about the COVID-19 lockdown ban in South Africa. We have added the below comment in the discussion to address this issue as best as possible without going off the paper’s focus and hope it satisfies the reviewer.

*While there are arguments for and against the use of electronic cigarettes as a harm reduction tool among scientists, the arguments in the South African media did not go deeper than the mention of a call to the government to exclude electronic cigarettes from the lockdown tobacco ban.* (page 23)

PLOS
While revising your submission, please upload your figure files to the Preflight Analysis and Conversion Engine (PACE) digital diagnostic tool, [https://pacev2.apexcovantage.com/](https://protect-za.mimecast.com/s/3ogwCLg1XDINl06RSr0R3m). PACE helps ensure that figures meet PLOS requirements. To use PACE, you must first register as a user. Registration is free. Then, login and navigate to the UPLOAD tab, where you will find detailed instructions on how to use the tool. If you encounter any issues or have any questions when using PACE, please email PLOS at [figures@plos.org](mailto:figures@plos.org). Please note that Supporting Information files do not need this step.

**Authors’ response:** A PACE version of the figures has been uploaded. Thank you.
