# Supplementary material for: “Are they trying to control us people?”: News media coverage of COVID-19 lockdown tobacco sales ban in South Africa
Source: PLoS One. 2022 Dec 12;17(12):e0278888. doi: 10.1371/journal.pone.0278888 (PMC9744309; doi:10.1371/journal.pone.0278888)
Supplement: Attachment — Submitted filename: Response to the reviewers_25Oct22.docx [file pone.0278888.s004.docx]

**Response to the reviewers**

**Journal Requirements:**

**Please review your reference list to ensure that it is complete and correct. If you have cited papers that have been retracted, please include the rationale for doing so in the manuscript text, or remove these references and replace them with relevant current references. Any changes to the reference list should be mentioned in the rebuttal letter that accompanies your revised manuscript. If you need to cite a retracted article, indicate the article’s retracted status in the References list and also include a citation and full reference for the retraction notice.**

Authors: We note that this comment is coming for the second time even after we have duly responded in our previous version. Kindly note that we have not used any retracted paper in our manuscript. We wish for the editor to mention the paper he or she thinks or knows has been retracted so that we can remove such paper(s) accordingly.

The retracted article of Stanton Glantz referred to by the reviewer is: *Bhatta DN, Glantz SA. Electronic Cigarette Use and Myocardial Infarction Among Adults in the US Population Assessment of Tobacco and Health. J Am Heart Assoc. 2019;8:e012317. DOI: 10.1161/JAHA.119.012317.* Please see link to retraction statement <https://www.ahajournals.org/doi/10.1161/JAHA.119.014519>

In none of the submitted versions of our paper have we mentioned or cited this article. The only two articles co-authored by Prof Stanton Glantz cited in our paper are:

*39. Crosbie E, Sebrié EM,* ***Glantz SA****. Tobacco industry success in Costa Rica: the importance of FCTC article 5.3. Salud publica de Mexico. 2012;54(1):28-38.*

*40.	Crosbie E, Sebrié EM,* ***Glantz SA****. Strong advocacy led to successful implementation of smokefree Mexico City. Tob Control. 2011;20(1):64-72.*

These papers have nothing to do with e-cigarettes or harm reduction. Is the editor also calling for these articles to not be used because they were co-authored by Stan Glantz?

**6. Review Comments to the Author**

**Please use the space provided to explain your answers to the questions above. You may also include additional comments for the author, including concerns about dual publication, research ethics, or publication ethics. (Please upload your review as an attachment if it exceeds 20,000 characters)**

**Reviewer #2: While the added sentence is somewhat responsive about the harm continuum and electronic or non combusted nicotine products -- the authors continue to state that all the concern about exempting electronic cigarettes is industry misinformation aboyt harm reduction . Thus it does not adequately address the issue that scientific opinion also provides scientific support for harm reduction and the substitution of much less harmful nicotine products cigarettes can save lives. The sentence could be acceptable - although its minimal - if several credible references in support of the science to date were cited , perhaps the least controversial of these are the following two citations (Public Health England latest scientific update and the very conservative review by 15 presidents of the worlds leading research society on nicotine and tobacco (SRNT). (The issue of Glantz and a personal vendetta is a distraction from the authors addressing adequately the real issue in their discussion and their own perhaps unintended bias in their interpretations of their own results -- as all evil industry related ). The issue is to take the highest ground of scientific evidence about harm reduced products and call for media accuracy based on science not industry past behavior ( and to indicate that despite the obvious and despicable industry tactics in general - which I agree with -- in terms of the industry being opposed to the entire ban etc, BUT as the authors stated -- one needs more press coverage with accurate science as well to counter industry tactics that do not protect public health but profits and their more massive budgets as the authors state. BUT again there IS ALSO scientific evidence (NOT ALL Industry misinformation) about the value and evidence in support of exempting electronic cigarettes / non combustible nicotine -- and correct misinformation in the media about a harm continuum of products -- so as to accelerate e and informed consumer decision making and government policy -- to save more smokers lives more quickly . So that can be clarified a bit mor strongly in the added sentence AND alos please ADD the two references below. These are from most credible sources and a full review of all the evidence to date . They qualify the weak and still therefore misleading added sentence from the authors -- one that suggests there are equal pro and con arguments for and against e-cigarettes (not a continuum of harm) even from a science point of view. This is not true ( i.e. thus by implication e-cigs are not less harmful and that any harm reduction claim is still to be ignored as its all part of an industry misinformation campaign. Harm reduction is not ONLY an industry plot, it is more and more strongly scientifically supported. I agree there was not enough in depth coverage of e-cigarettes as stated in the revision , but it does not clarify the core issue to ensure accurate media coverage of the harm continuum and reduced harm products to save lives. The authors can do better at composing a few sentences than their current added sentence.**

**1. Balancing Consideration of the Risks**

**and Benefits of E-Cigarettes. David J. K. Balfour, DSc, Neal L. Benowitz, MD, Suzanne M. Colby, PhD, Dorothy K. Hatsukami, PhD, Harry A. Lando, PhD,**

**Scott J. Leischow, PhD, Caryn Lerman, PhD, Robin J. Mermelstein, PhD, Raymond Niaura, PhD, Kenneth A. Perkins, PhD, Ovide F. Pomerleau, PhD, Nancy A. Rigotti, MD, Gary E. Swan, PhD, Kenneth E. Warner, PhD, and Robert West, PhD.**

**Am J Public Health. Published online ahead of print**

**August 2021:e1–e12. https://doi.org/10.2105/AJPH.2021.306416)**

**2. McNeill A, Brose LS, Calder R, Simonavicius E,**

**Robson D. Vaping in England: An Evidence Update**

**Including Vaping for Smoking Cessation, February**

**2021. A Report Commissioned by Public Health**

**England. Public Health England. 2021. Available**

**at: https://assets.publishing.service.gov.uk/**

**government/uploads/system/uploads/**

**attachment_data/file/962221/Vaping_in_**

**England_evidence_update_February_2021.pdf.**

**Accessed March 6, 2021.**

**Authors’ response:** We still believe that our paper is not the place to make a case for harm reduction or not, as we only aimed to objectively present what local media reported about the COVID-19 lockdown ban in South Africa.

We totally respect the reviewer’s stance on e-cigarettes as a “harm reduction” tool. However, we believe the jury is not out yet on whether e-cigarettes are the “safer options” for cigarettes smokers and indeed the second reference by McNeil et al. (2021) suggested by the reviewer says, “The COT also suggested people who had not smoked tobacco but vaped would likely experience some adverse health effects.” This report by McNeil et al on vaping and smoking cessation does not actually answer the questions we have raised in our response to date about the fact that switching from one addictive substance to another addictive substance with a potential for harm does not qualify as “harm reduction”. Also, the report noted adverse health effects reported by users which were perceived to be associated with vaping. The first reference by Balfour et al. (2021) is also an opinion piece which does not differ from the discussions we have been having on this topic.

It is worthy of note that in South Africa, we have not found e-cigarettes to help smokers quit. We cannot recommend e-cigs for smoking cessation in South Africa when our own data show otherwise. Please see below citation to a paper co-authored by the lead author of this manuscript.

Agaku, I., Egbe, C. O., & Ayo-Yusuf, O. (2022). Associations between electronic cigarette use and quitting behaviours among South African adult smokers. *Tobacco Control*, *31*(3), 464-472.

Lastly, we are still worried that the reviewer is requiring us to support his own stance on e-cigarettes as a harm reduction tool on a paper that is NOT explicitly focused on harm reduction. We believe this paper is not the place to make this argument as we have indicated in previous versions of our manuscript that the discussion in the media did not focus on these issues being debated by the reviewer. It would be absurd to turn the discussion section into an ‘e-cigs are harm reducing or not” argument when our results do not lend themselves to that debate. Thank you.

**PLOS
While revising your submission, please upload your figure files to the Preflight Analysis and Conversion Engine (PACE) digital diagnostic tool,** [**https://pacev2.apexcovantage.com/**](https://protect-za.mimecast.com/s/3ogwCLg1XDINl06RSr0R3m)**. PACE helps ensure that figures meet PLOS requirements. To use PACE, you must first register as a user. Registration is free. Then, login and navigate to the UPLOAD tab, where you will find detailed instructions on how to use the tool. If you encounter any issues or have any questions when using PACE, please email PLOS at** [**figures@plos.org**](mailto:figures@plos.org)**. Please note that Supporting Information files do not need this step.**

**Authors’ response:** A PACE version of the figures has been uploaded. Thank you.
